# Transient reprogramming primes the heart for repair

**DOI:** 10.20517/jca.2021.31

**Published:** 2022-01-01

**Authors:** Natalie A. Gude, Fareheh Firouzi, Mark A. Sussman

**Affiliations:** SDSU Heart Institute and Biology Department, San Diego State University, San Diego, CA 92182, USA.

## Abstract

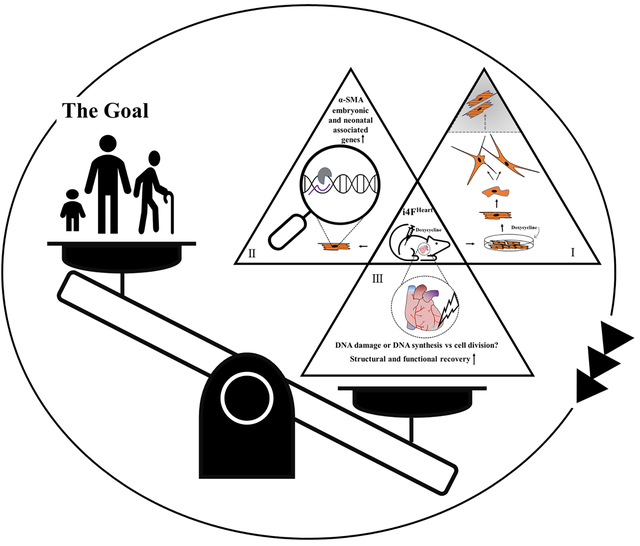

Cardiomyocyte loss followed by scar formation is the leading cause of heart failure upon pathological injury, chronic or acute stress, and aging. Despite decades of investigation, therapeutic development to enhance cardiomyocyte turnover and restore the structural and functional integrity of the heart has been limited due in part to the inherent lack of proliferative capacity within adult cardiomyocytes. A therapeutic solution to the conundrum of post-mitotic cardiomyocytes has long been sought after, leading to a range of pre-clinical interventional approaches including (but not limited to) overexpression of cell cycle proteins, induction of systemic hypoxemia, as well as transcriptional and hormonal regulation. The consensus outcome reveals dedifferentiation rather than proliferation potential of adult mammalian cardiomyocytes based upon phenotypic changes such as (1) loss of myofibrillar structure; (2) expression of stem cell markers; and (3) exhibition of immature metabolic functions^[1]^. In retrospect, the yet to be resolved refractory nature of adult cardiomyocytes with respect to cell cycle progression and cell division necessitates the need for an innovative approach to promote adult cardiomyogenesis.

Cellular reprogramming via the four pluripotency factors, OCT4, SOX2, KLF4, and MYC (OSKM), is a promising approach to epigenetically remodel somatic cells of various lineages into an induced pluripotent stem cell state. Partial reprogramming (PR) via the transient introduction of OSKM reverses age-associated phenotypes and promotes the regenerative potential of adult tissues such as skeletal muscle^[2]^. In an effort to illustrate the myocardial therapeutic significance of PR, Chen *et al*.^[3]^, demonstrated that short-term myocardial introduction of OSKM reprograms cardiomyocytes to a fetal phenotype evidenced by (1) transcriptome resembling neonatal and embryonic developmental stages; (2) expression of α-SMA; and (3) morphological rearrangement and cell cycle re-entry *in vitro*. Regardless of the time of induction, *in vivo* OSKM expression promoted structural recovery of the heart following myocardial infarction; however, functional results varied among induction regimens.

Driving cell cycle progression to achieve true proliferation in adult mammalian cardiomyocytes represents a longstanding goal in the ongoing effort to repair damaged myocardium^[4]^. From cardiomyocyte-specific overexpression of cyclins to transdifferentiation of fibroblasts into myocytes, to differentiating induced pluripotent stem cells (iPSCs) into cardiomyocytes, time and again, the results indicate that such approaches are unlikely to translate directly into clinically relevant therapies. And yet, each new insight into cardiomyocyte biology increases the knowledge base necessary to design better informed regenerative therapies. The current findings by Chen *et al*.^[3]^ reveal a threshold of cardiomyocyte dedifferentiation in adult mice, after which cardiac function declines and cardiac tumors form, in contrast to lower vertebrates and neonatal mammals, which tolerate greater cardiac plasticity.

Creating pre-clinical models of cardiac repair that reflect clinically therapeutic scenarios remains an ongoing challenge in cardiac research. Differentiation of cardiac progenitor cells, iPSCs, or reprogrammed cardiomyocytes into functional, mature myocytes remains a major roadblock for translational applications. Experimental treatments that coincide with myocardial injury tend to represent cardioprotective salvage models rather than therapies that replace damaged myocardium post-injury. Functional outcomes may be improved; however, cardiac patients are unlikely to receive regenerative treatment at the time of a cardiac event. Interestingly, the infarction injury results presented by Chen *et al*.^[3]^ highlight the challenge of associating clinically relevant cardiac repair with endogenous cardiomyocyte cell cycle progression. All of the partial reprogramming regimens result in reduced scar size in infarcted hearts, and post-injury therapeutic treatment yields the most 5-Ethynyl-2′-deoxyuridine (EdU) + cardiomyocytes. However, the best functional outcomes are observed in the pre-injury treatment group, whereas no functional improvement is observed in the post-injury therapeutic treatment cohort. Salvage, cell cycle progression, and function appear disconnected in this repair model.

Identifying true cardiomyocyte replication represents another point of contention among researchers. Mature cardiomyocytes are generally post-mitotic; however, their nuclei can undergo changes that appear to reflect proliferation, such as DNA synthesis in response to stress, binucleation or karyokinesis without cytokinesis, or increases in ploidy during aging. Measures of cell cycle re-entry such as EdU, phospho histone H3, even aurora B kinase, are insufficient to definitively prove full cardiomyocyte cellular division^[5]^. Reagents that track cell cycle in real-time, such as various transgenic fluorescence ubiquitination cell cycle indicator reporter animal models, can provide dynamic readouts of cell cycle status *in vitro* and *in vivo*. Perhaps the most convincing data demonstrating cardiomyocyte proliferation in the current publication are live imaging videos tracking tagged cells going through karyo and cytokinesis. Of course, the next crucial step in the regenerative process is to convert these dedifferentiated reprogrammed proliferating cardiomyocytes into functional adult cells that contribute to cardiac function. Whether comparable division and re-differentiation occur in damaged reprogrammed hearts remains to be demonstrated.

Systemic context plays a crucial role in myocardial regenerative potential. Metabolic and paracrine factors, and the state of the extracellular matrix all have an enormous impact on cardiomyocyte cell cycle activity^[6–8]^. Reprogrammed cardiomyocytes in an adult heart are not equivalent to developing cardiomyocytes maturing in concert with associated systemic postnatal hormonal changes^[9]^. Studies have shown that the paracrine, metabolic and structural milieu of an adult mammalian heart favor cellular hypertrophy or senescence over cardiomyocyte proliferation^[10]^. The myocardial environment in most heart disease patients is probably even less conducive to proliferation and repair, underscoring the challenge of transforming reprogrammed cells into new functional new myocytes. Nonetheless, while the gap between basic research findings and clinically relevant cardiac therapies remains unbridged, cumulative research findings are essential to successful development of those therapies.

Many questions about cardiomyocyte proliferation remain unanswered from the basic and clinical perspectives, perhaps the most important being: can adult human cardiomyocytes be coaxed to divide in response to injury on a therapeutically relevant level, and is it possible to quantify this proliferation in recipient human patients? Experiments performed in young adult mice in no way reflect the systemic profile of human heart patients, many of whom are elderly or have underlying comorbidities such as hypertension, diabetes, or other metabolic dysregulation. Timing and dosage of a hypothetical reprogramming therapy, in light of the results presented by Chen *et al*.^[3]^, represent substantial translational hurdles. How can cardiomyocyte reprogramming be administered safely and titrated to avoid the deleterious effects observed in over-reprogrammed hearts? Would this be possible given the enormous variability among human cardiac patients? Perhaps the real takeaway lesson from this and similar studies investigating cardiomyocyte proliferation lies in understanding what makes an adult cardiomyocyte healthy and capable of cell cycle re-entry to the point of replication. From a preventative medicine point of view, a more realistic approach may be to identify lifestyles and habits that support the more youthful and proliferative cardiomyocyte phenotypes identified in partial reprogramming models. Transcriptomic and proteomic profiling studies comparing youthful and diseased or senescent cardiac phenotypes may provide therapeutically relevant targets for suppressing senescence-associated secretory phenotype, inflammation, or oxidative stress in a time and a dose-dependent way to encourage myocardial healing. Evaluating signaling pathways associated with youthful or proliferative phenotypes could also reveal safer, more reliable targets. Ultimately, combinatorial therapies incorporating multiple restorative strategies probably hold the most promise for improving cardiac patient outcomes.

In summary, the holy grail of physically mending damaged human hearts remains elusive. Considerable time, resources, and effort have gone toward understanding myocardial injury, repair, aging, and possible regeneration in vertebrate animal models. Approaches including cell therapy, tissue engineering, and driving adult mammalian cardiomyocytes to proliferate in response to damage as documented in neonatal hearts, or adult fish and reptile systems, have yielded valuable new insights into cardiac biology. Moreover, although a marketable therapy to repair pathologically challenged human hearts has not yet materialized, this goal has inspired incredible scientific creativity and a deeper understanding of cardiac cellular and molecular biology. Put another way, research findings cannot be valued solely on their immediate translational relevance, or unduly influenced by increasing pressure to produce therapies and cures. However, clinically driven studies that build on collective basic and translational research findings bring cardiac medicine closer to curing or even preventing heart disease altogether.
